# Additive and Subtractive Scrambling in Optional Randomized Response Modeling

**DOI:** 10.1371/journal.pone.0083557

**Published:** 2014-01-08

**Authors:** Zawar Hussain, Mashail M. Al-Sobhi, Bander Al-Zahrani

**Affiliations:** 1 Department of Statistics, Quaid-i-Azam University, Islamabad, Pakistan; 2 Department of Mathematics, Umm Alqura University, Makkah, Saudi Arabia; 3 Department of Statistics, King Abdulaziz University, Jeddah, Saudi Arabia; University of Rochester, United States of America

## Abstract

This article considers unbiased estimation of mean, variance and sensitivity level of a sensitive variable via scrambled response modeling. In particular, we focus on estimation of the mean. The idea of using additive and subtractive scrambling has been suggested under a recent scrambled response model. Whether it is estimation of mean, variance or sensitivity level, the proposed scheme of estimation is shown relatively more efficient than that recent model. As far as the estimation of mean is concerned, the proposed estimators perform relatively better than the estimators based on recent additive scrambling models. Relative efficiency comparisons are also made in order to highlight the performance of proposed estimators under suggested scrambling technique.

## Introduction

To procure reliable data on stigmatizing characteristics, Warner [1] introduced the notion of randomized response technique where the respondent himself selects randomly one of the two complementary questions on probability basis. Greenberg et al. [2] extended the Warner's [1] work to collect the data on quantitative stigmatizing variables. Since then, several authors have worked on quantitative randomized response models including, Eichhorn and Hayre [3], Gupta and Shabbir [4], Gupta and Shabbir [5], Bar-Lev et al. [6], Gupta et al. [7], Hussain and Shabbir [8], Saha [9], Chaudhuri [10], Hussain and Shabbir [11] and references therein. Quantitative randomized response models are classified into fully (Eichhorn and Hayre [3]), partial (Gupta and Shabbir [5]), Bar-Lev et al. [6]) and optional randomized response models (Gupta et al. [4]), Gupta et al. [7], Huang [12]). In a fully randomized response models all the responses are obtained as scrambled responses. In a partial randomized response model a known proportion of respondents is asked to report their actual responses while the others report scrambled responses.

Our focus in this article is on ORRMs only. The notion of ORRM started with Gupta et al. [4]. The concept of ORRM is based on the respondent's perception about sensitivity of the variable of interest. Using ORRM, a respondent can report the truth (or scramble his/her response) if he/she perceives the study variable as non sensitive (sensitive) to him/her. The proportion of respondents reporting the scrambled response is unknown, and is termed as the sensitivity level of the study variable. Gupta et al. [4] used multiplicative ORRM and provided unbiased (biased) estimator of mean (sensitivity). Moreover, Gupta et al. [4] ORRM requires approximation in order to derive the variances of the estimators. In Gupta et al. [4] ORRM, simultaneous estimation of mean and sensitivity is not possible. To avoid approximation, Gupta et al. [7], Huang [12], Gupta et al. [13] and Mehta et al. [14] proposed ORRMs to provide unbiased estimators of mean and sensitivity level. Gupta et al. [7] and Huang [12] are the one-stage ORRMs, Gupta et al. [13] is a two-stage ORRM whereas Mehta et al. [14] is a three-stage ORRM. Gupta et al. [7], Gupta et al. [13] and Mehta et al. [14] used additive scrambling whereas Huang [12] used a linear combination of additive and multiplicative scrambling. Further, Gupta et al. [15] observed that additive scrambling yields more precise estimators than a linear combination of additive and multiplicative scrambling by Huang [12]. Also, Gupta et al. [16] observed that in Gupta et al. [13] two-stage ORRM a large value of truth parameter (*T*) is required when the study variable is highly sensitive. Motivated by the advocacy of additive scrambling and requirement of larger value of truth parameter (*T*), Mehta et al. [14] proposed a three stage ORRM by introducing a forced scrambling parameter (*F*). Mehta et al. [14] established the better performance of estimator of mean but did not discuss the performance of sensitivity estimator. As far as the estimation of mean is concerned, Mehta et al. [14] ORRM can be further improved by using a multi-stage randomization but it results in a poor estimation of sensitivity level.

All of the ORRMs mentioned above share a common feature of splitting the total sample into two subsamples. We base our proposals on two strategies: (i) taking two subsamples and making use of additive scrambling in one subsample and subtractive scrambling in the other, and (ii) drawing a single sample and collecting two responses from each respondent through additive and subtractive scrambling. Through our strategies, we plan to improve Mehta et al. [14] ORRM for estimating the mean. As far as estimation of mean is concerned, we show that the proposed ORRM is better than Mehta et al. [14], Huang [12] and Gupta et al. [13] ORRMs. We show that there is no need of large value of the parameter (*T* or *F*) when the study variable is either low, moderately or highly sensitive. In addition, we also propose an estimator of the variance of the study variable.

We now briefly discuss three of the background ORRMs, namely, the Mehta et al. [14], Huang [12] and Gupta et al. [13].

### Mehta et al. [14] ORRM

Assume that the interest lies in unbiased estimation of the mean 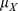 and the sensitivity level 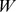 of the study variable 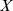. Let 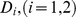 be the unrelated scrambling variable. Two independent subsamples of size 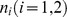, are drawn from the population through simple random sampling with replacement such that 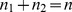, the total sample size required. In 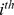 subsample, a fixed predetermined proportion 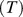 of respondents is instructed to tell the truth and a fixed predetermined proportion 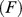 of respondents is instructed to scramble additively their response as 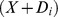. The remaining proportion 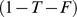 of respondents have an option to scramble their response additively if they consider the study variable sensitive. Otherwise, they can report the true response 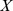. Let 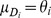, be the known mean, and 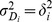, be the known variance of the positive-valued random variable 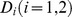. The optional randomized response from 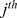 respondent in the 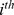 subsample is given by:
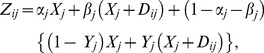
(1)where 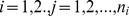, 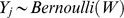, 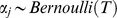 and 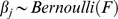. The expectation of the sample response 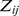 from 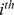 sample is given by:
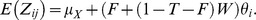
Taking 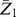 and 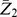 as the observed means from the two subsamples, Mehta et al. [14] proposed the following estimators of 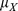 and 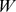.
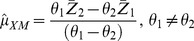
(2)

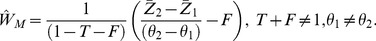
(3)The variances of estimators in (2) and (3) are given by:
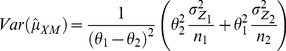
(4)

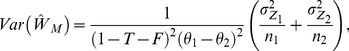
(5)where
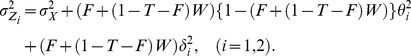
(6)


### Gupta et al. [13] ORRM

It is interesting to note that for 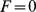, the Mehta et al. [14] ORRM reduces to Gupta et al. [13] ORRM. . Let 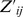 be the optional scrambled response from 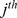 respondent in the 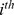 subsample then taking 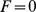 in (1)–(5), unbiased estimators and their variances are given by:
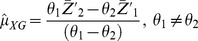
(7)

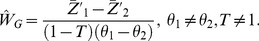
(8)

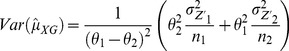
(9)

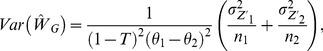
(10)where
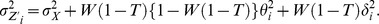



### Huang [12] ORRM

Each respondent in the 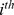 subsample is provided with two randomization devices which generate two independent random variables, say 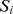 and 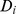, from some pre-assigned distributions. The respondent chooses randomly by himself one of the following two options: (a) report the true response 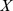 (if you do not feel the study variable sensitive), or (b) report the scrambled response 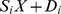 (if you feel the study variable sensitive). Let 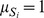, be the known mean, and 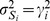, be the known variance of the positive-valued random variables 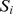. The optional randomized response 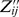 from 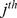 respondent in the 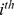 subsample is given by:
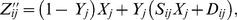
(11)The expectation of sample response 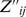 from 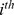 sample is given by:
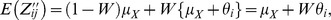
since 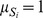. Huang [2] proposed the following estimators of 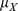 and 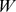.
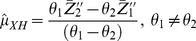
(12)

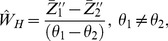
(13)where 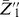 and 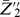 are the observed means from the two subsamples. The variances of estimators in (12) and (13) are given by:
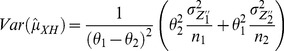
(14)

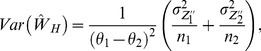
(15)where
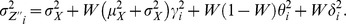



## Proposed Procedures

In this section, we propose split sample and double response approaches using Mehta et al. [14] ORRM.

### Split sample approach

Unlike Mehta et al. [14], in the proposed procedure, we use an additive scrambling in one subsample and subtractive scrambling in the other. All the other procedure is same as that of Mehta et al. [14]. Let 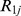 and 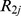 be response from 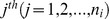 respondent selected in the 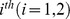 sample, then 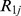 and 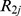 can be written as:
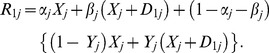
(16)

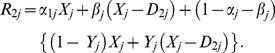
(17)The expected responses from the two subsamples are given by:
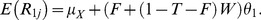
(18)

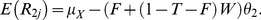
(19)Solving (18) and (19), we get:
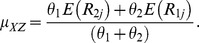


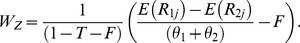
Estimating 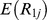 and 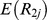 by the respective sample means 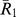 and 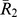, unbiased estimators of 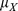 and 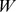 are proposed as:
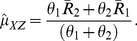
(20)

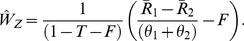
(21)Unbiasedness of 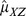 and 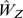 can be easily established through (18) and (19). The variances of 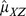 and 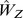 are given by :
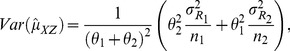
(22)

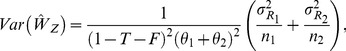
(23)where 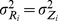.

It is important to note that subtractive scrambling in the second subsample is same as the additive scrambling if 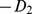 is viewed as the new scrambling variable. We anticipate two advantages by calling it subtractive scrambling. Firstly, it is easier just to subtract a constant (randomly chosen by the respondent) from the actual response on sensitive variable. Second advantage is a psychological one in nature. Perhaps, due to social desirability, a typical respondent would like to report smaller response in magnitude. In other words, respondents would be happy in underreporting, in general. Thus, subtracting a positive constant from the actual response would help satisfying the social desirability of underreporting. Of course, these two advantages are gained in the second subsample only since 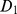 and 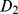 are positive valued random variables. On average, affect of additive scrambling in one subsample is offset by subtractive scrambling in the other. As a result, parameters are estimated with increased precision.

**Theorem 2.1:** For 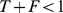, 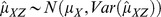 and 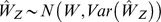.

**Proof:** Since 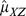 and 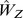 are the linear combinations of sample means, application of central limit theorem gives the required result.

In view of the fact that 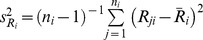 is an unbiased estimator of 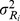 we have the following theorems.

**Theorem 2.2:** An unbiased estimator of 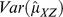 is given by:
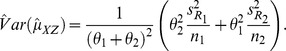



**Theorem 2.3:** An unbiased estimator of the 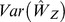 is given by:
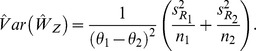



**Proofs:** The proofs of the above Theorems (2.2 and 2.3) can easily be provided by utilizing the fact that 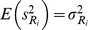.

**Theorem 2.4:** An unbiased estimator of 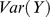 is given by:
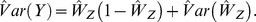



**Proof:** Applying the expectation operator at 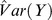, we get:
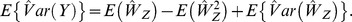
Then, applying Theorem 2.3, we get:
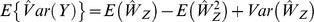


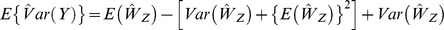


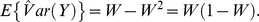
Now, we consider the estimation of variance 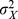 of the sensitive variable 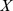. Provided that 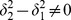, from (6) we can, after a simple algebra, write that
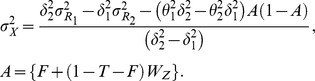
We define unbiased estimators of 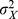 in the following theorems.

**Theorem 2.5:** In case when 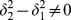, an unbiased estimator of 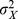 is given by:
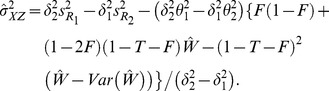
(24)


**Theorem 2.6:** In case when 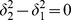, an unbiased estimator of 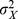 is given by:
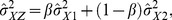
(25)where 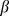 is known constant belonging to the interval 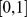, 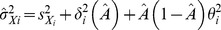 and 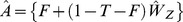.

**Proofs:** The above Theorems 2.5 and 2.6 can be proved by noting that 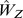 and 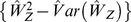 are unbiased estimators of 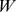 and 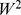 respectively. Taking expectation of (24) and (25), we get 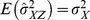.

### Double response approach

Without incurring any additional sampling cost, Mehta et al. [14] ORRM may also be improved by taking two responses from each respondent. We take scrambling variables the same as defined in Mehta et al. [14] ORRM. To report the first (second) response, respondents are requested to use additive (subtractive) scrambling with the variable 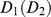. Let 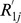 and 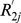 be the two responses of 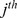 respondent then the two responses can be written as
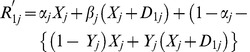
(26)

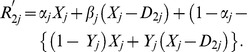
(27)


It is obvious from (26) and (27) that the true value of sensitive variable 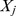 cannot be worked out for the respondents feeling study variable sensitive enough. The reported responses of a particular respondent would be same if he/she feels study variable insensitive. In this case, he/she reports true value of study variable both the times. This is not challenging since the respondents feeling study variable insensitive would be willing to dispose their true value on sensitive variable. Thus, it may be concluded that privacy of respondents, feeling study variable sensitive, remains intact. As correctly pointed out by one of the referees, there is extra burden on the respondent if he/she has to report twice. This issue may be tackled by explaining whole the procedures to the respondent before actually obtaining data. He/she must be assured that his/her actual response on sensitive variable cannot be traced back to his/her actual response. Further he/she must be made clear that interest of the study lies in the estimation of parameters only. Moreover, we do not need any additional sampling cost to obtain two responses. Thus, obtaining two responses from a respondent should not be an issue in a particular study.

The expected responses from the 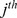 respondent are same as given by (18) and (19). Thus 
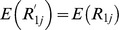
 and 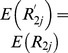. This implies that unbiased estimators of 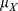 and 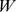 may be suggested as:
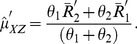
(28)

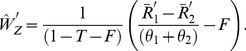
(29)The variances of 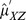 and 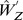 are given by :
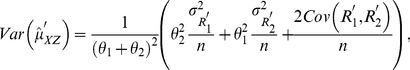
(30)

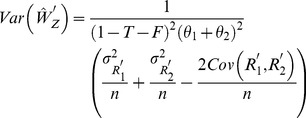
(31)where
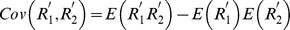


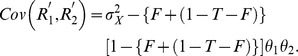



In some studies, interest of researchers lies in estimating 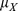
rather than the sensitivity level 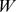 of variable 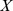 while it is of major interest in other studies. Following Huang [12], we define a linear combination of 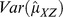 and 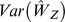 in order to find the optimum allocation of sample size. Thus, depending upon the interest of researchers, optimum subsample sizes can be obtained.

Consider,
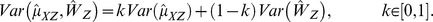
Using Lagrange approach to minimize 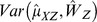 under the restriction that 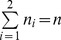, we get:
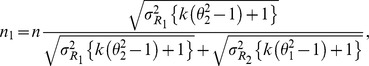
and
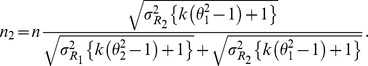
With these optimum sample sizes, the minimum value of 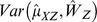 is given by:
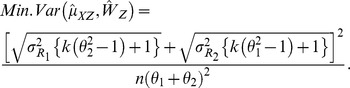



In practice, 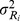 is unknown and the optimum allocation of sample sizes cannot be made. Following Murthy [17], the unknown values of 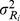 can be estimated from pilot surveys, past experience or simply an intelligent guess can be made about 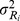.

## Privacy Protection Discussion

There are many privacy measures suggested by different authors. We take 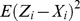 as the measure of privacy. This measure of privacy is proposed by Zaizai et al. [18]. A given model is taken as more protective against privacy if 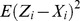 is higher. For a model providing privacy protection to some extent 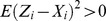. On the other hand, if a model does not provide any privacy 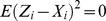. For a given model, the larger the 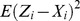, the larger the privacy provided by the model.

The measures of privacy for Mehta et al. [14] ORRM are given by 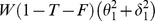 and 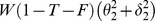 in the first and second subsamples, respectively. Similarly for Gupta et al. [13] model it is 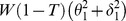 in the first sample, and 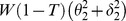 in the second sample. This shows that, in both the subsamples, Gupta et al. [13] ORRM is more protective compared to Mehta et al. [14] ORRM. The measures of privacy for Huang [12] ORRM are given by 
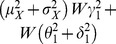
 and 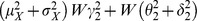 in the first and second subsamples, respectively. The measures of privacy for the proposed estimator in split sample approach are the same as that of Mehta et al. [14] ORRM. In double response approach the measure of privacy is given by 
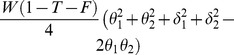
 which is equal to measure of privacy provided by Mehta et al. [14] ORRM if and only if 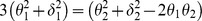 or 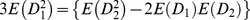. This shows that the proposed double response approach may be made more protective compared to Mehta et al. [14] ORRM at the cost of increased variance. In fact, it is a trade-off between the efficiency and privacy protection. That is, we can have highly efficient estimator by compromising on privacy. Similarly, we can build a more protective model by compromising on the efficiency.

## Efficiency Comparison

We compare the proposed split sample and double response approaches with the Mehta et al. [14], Huang [12] and Gupta et al. [13] ORRMs in terms of relative efficiency.

### (i) 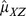 versus 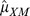 and 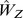 versus 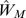


The proposed estimators 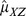 and 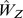 are relatively more efficient than the corresponding estimators 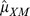 and 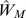 of Mehta et al. [1] if 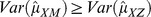 and 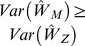. Since 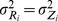, from (4), (5), (20) and (21), it is easy to show that 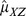 and 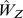 are relatively more efficient than 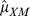 and 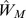 if
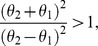
which is always true for every value of 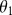 and 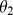.

### (ii) 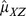 versus 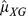 and 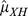


**Figure 1:**
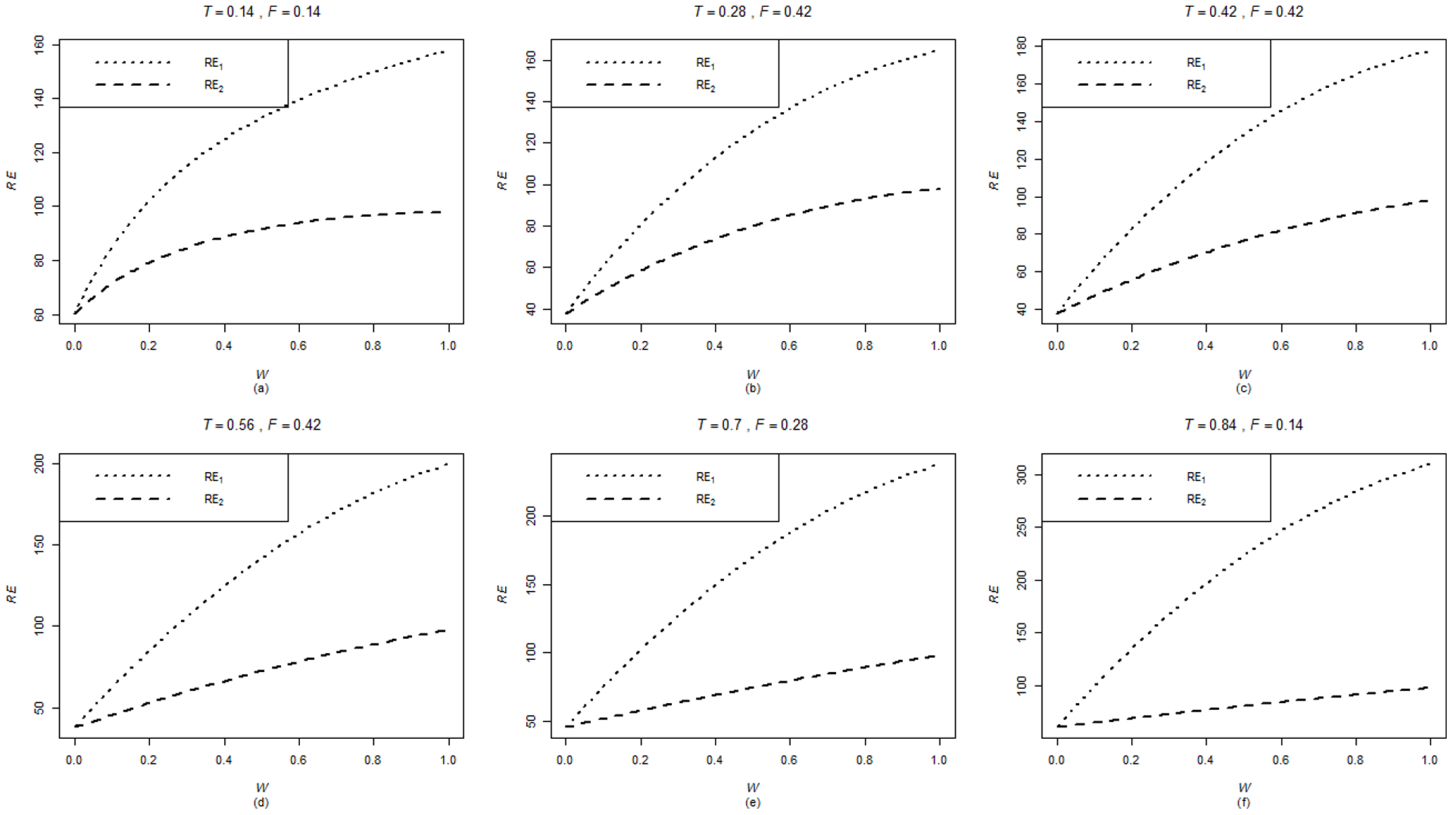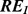 and 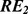 for 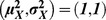, 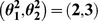, 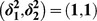 and 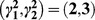.

**Figure 2:**
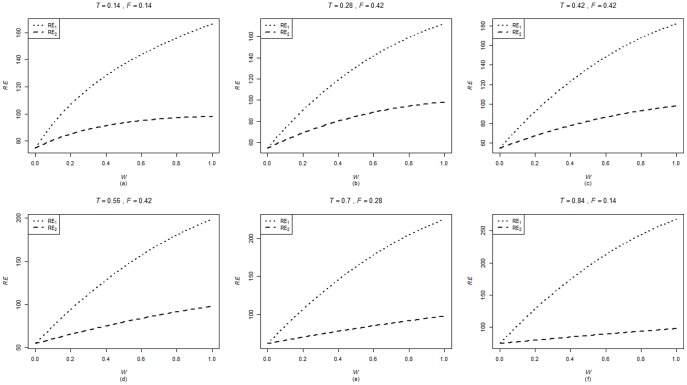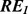 and 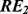 for 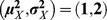, 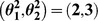, 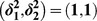 and 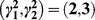.

**Figure 3:**
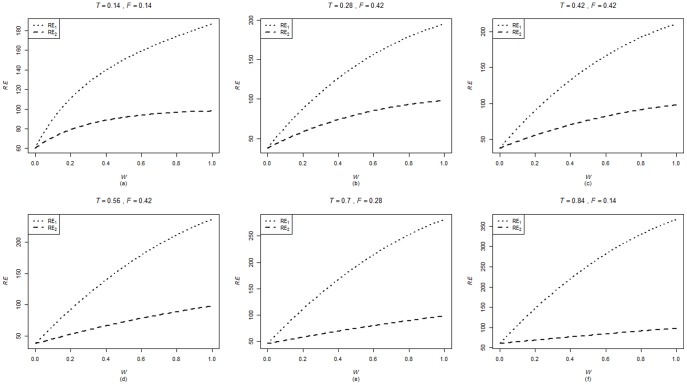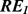 and 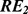 for 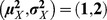, 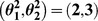, 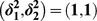 and 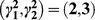.

**Figure 4:**
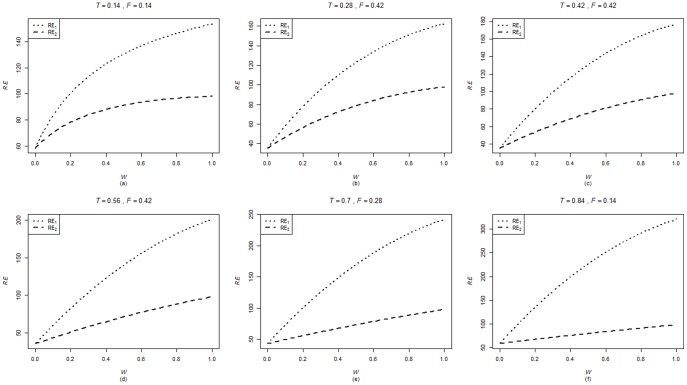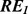 and 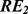 for 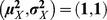, 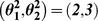, 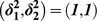 and ,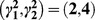.

The proposed estimator 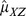 is relatively more efficient than 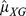 and 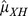 if 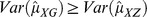 and 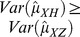. From (9), (14) and (21), we see that it is difficult to derive the efficiency conditions for 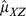. We calculated the relative efficiency numerically through simulations by defining 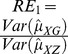 and 
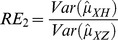
. For a simulation study, we fixed 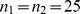. We assumed that 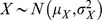, 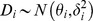 and 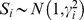, 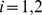. To simulate the data from the first subsample, we generated 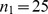
values from a Bernoulli variable, say 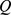, with the parameter 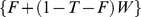, where 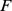, 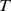 and 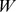 are known. We, then, generated 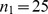 random values each on the variables 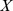 and 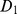 from 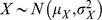 and 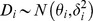, respectively. We took 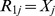 if 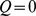, and 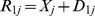, otherwise. Similarly, 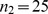 values of 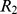 from the second subsample are generated as: 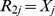 if 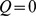, and 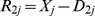, otherwise. Same algorithm is used to generate the values of 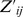 and 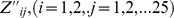. Once the data have been generated, different estimators 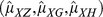 are computed using the corresponding formulae in (7), (12) and (20). The variances of these estimators are obtained using 5000 iterations. The relative efficiency results (for the different scenarios given below) are given in the Figures 1–4.

### (iii) 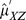 versus 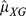 and 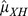


**Figure 5:**
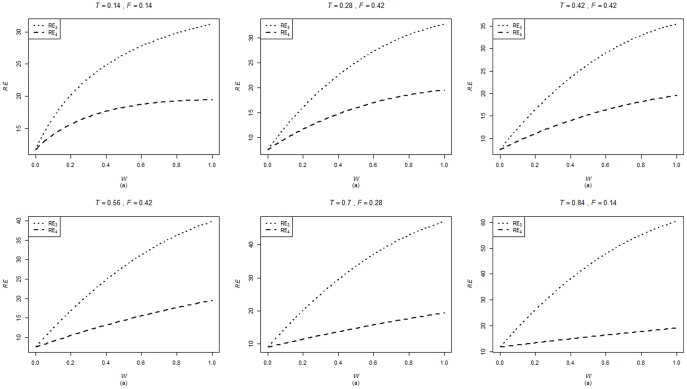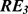 and 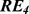 for 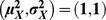, 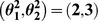, 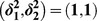 and,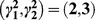.

**Figure 6:**
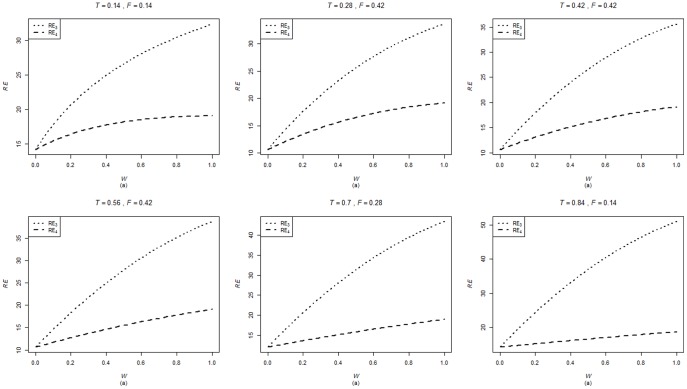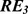 and 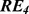 for 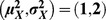, 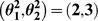, 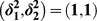 and 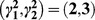.

**Figure 7:**
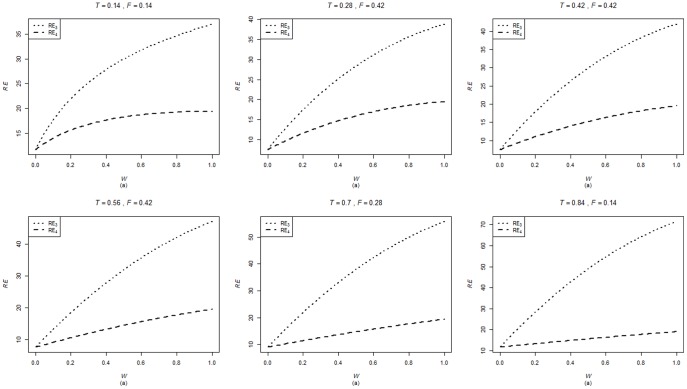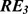 and 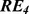 for 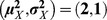, 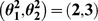, 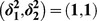 and 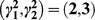.

**Figure 8:**
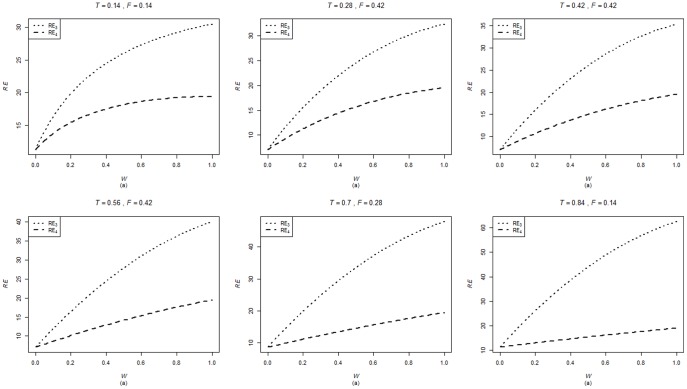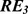 and 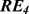 for 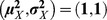, 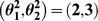, 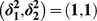 and 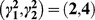.

The proposed estimator 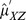 is relatively more efficient than 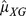 and 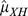 if 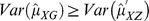 and 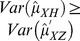. From (9), (14) and (30), we see that it is difficult to derive the efficiency conditions for 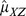. We, again, calculated the relative efficiency numerically through simulations by defining 
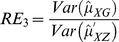
and 
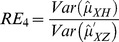
. We used the similar algorithm to simulate the values of 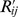, 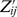 and 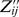. It is to be noted that we simulated 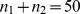 values of 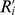 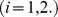 and 25 values each of 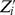 and 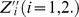. The relative efficiency results are given in the Figures 5–8.

To calculate 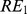, 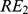 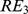 and 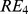,we take the following different scenarios:

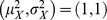
, 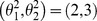, 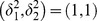, 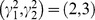

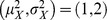
, 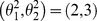, 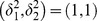, 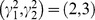

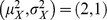
, 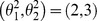, 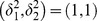, 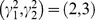

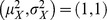
, 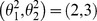, 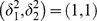, 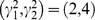,

and study the effect of 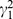 and 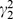 on 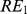, 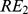, 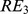 and 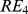. The relative efficiencies are calculated for different values of 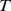 and 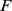 over the whole range of 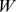. It is observed that the proposed estimator 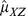 performs better (in terms of relative efficiency) than the 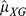 and 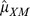. Also, the proposed estimator 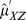 performs relatively better than 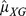 and 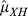. It can easily be verified through simulations that 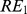, 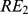, 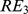 and 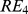 are independent of 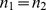. To save the space we have not presented the graphs for varying values of 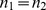. From Figures 1–8 following observations are made.

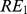
, 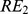, 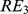 and 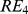 are not seriously affected by the difference between 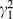 and 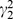 when the other parameters are fixed (see Figures 1 and 4 or 5 and 8).

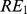
, 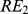, 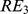 and 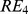 increase, over the whole range of 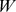, with an increase in 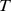 when the other parameters, except 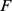, are kept fixed (see Figures 1–8).

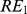
, 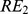, 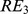 and 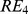 are not seriously affected by change in 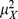 and/or 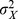 (see Figures 1 and 3, and 5 and 7 or 1 and 2 and 5 and 6).
- Split sample approach is more efficient than double response approach
- The proposed estimators of mean through split sample and double response approaches do not need a smaller values of 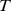 irrespective of the sensitivity level 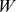 and the forced scrambling parameter 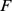.

## Conclusion

To estimate the mean, variance and the sensitivity level of a sensitive variable optional randomized response model by Mehta et al. [14] is improved. Utilizing the idea of additive scrambling in one sample and subtractive scrambling in the other subsample, we have proposed unbiased estimators of mean, variance and sensitivity level. We compared the proposed procedure with Mehta et al. [14] Huang [12], and Gupta et al. [13] procedure. The proposed idea resulted in the improved estimation of mean of the study variable. It has been shown by Huang [12] that his procedure works better than Gupta et al. [4] procedure. Therefore, the proposed split sample procedure is also better than Gupta et al. [4] procedure both in terms of relative efficiency and providing unbiased estimators of the mean 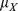, sensitivity level 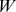 and variance 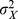 of the study variable. Like Huang [12], the proposed procedure has the same advantage of estimating the variance of 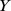 with no bias. Unlike Gupta et al. [4], proposed procedures do not require larger value of truth parameter 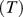 when the study variable is highly sensitive. This may be considered the major advantage of the proposed procedures. It has been established that the proposed procedure of estimating mean is more efficient than all the procedures considered in this study. Moreover, as far as, the estimation of sensitivity is concerned we observed that the proposed estimators are less efficient (not shown in the figures) than all the estimators considered here except Mehta et al. [14].

As a final comment, we recommend using proposed procedures in the field surveys without increasing sampling cost when estimation of mean of the study variable is of prime interest.
