# Correction: Additive and Subtractive Scrambling in Optional Randomized Response Modeling

**DOI:** 10.1371/journal.pone.0101308

**Published:** 2014-06-19

**Authors:** The PLOS ONE Staff

There are errors in the fifth paragraph under the “Double response approach” heading of the Proposed Procedures section of this article: The expressions for optimum sample sizes and optimum variance were derived incorrectly. The correct paragraph is:

Consider,
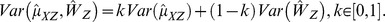



Using Lagrange approach to minimize 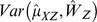 under the restriction that 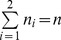, we get:
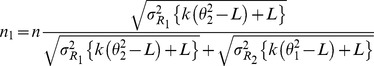
and
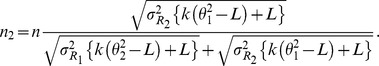



With these optimum sample sizes, the minimum value of 
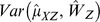
 is given by:
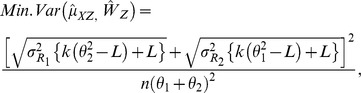
where 
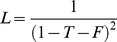
.
